# High-quality draft genome of the methanotroph *Methylovulum psychrotolerans* Str. HV10-M2 isolated from plant material at a high-altitude environment

**DOI:** 10.1186/s40793-018-0314-2

**Published:** 2018-04-12

**Authors:** Alejandro Mateos-Rivera, Tajul Islam, Ian P. G. Marshall, Lars Schreiber, Lise Øvreås

**Affiliations:** 1 Department of Biology, University of Bergen, Bergen, Norway; 2 Faculty of Engineering and Science, Western Norway University of Applied Sciences, Sogndal, Norway; 3 Center for Geomicrobiology, Department of Bioscience, Aarhus University, Aarhus, Denmark; 4 UNIS, the University Centre in Svalbard, Longyearbyen, Norway; 5 Present address: Energy, Mining and Environment, National Research Council, Montreal, QC Canada

**Keywords:** *Methylovulum psychrotolerans* HV10-M2, *Methylovulum*, *Gammaproteobacteria*, Methanotroph, High-altitude

## Abstract

Here we present the genome of *Methylovulum psychrotolerans* strain HV10-M2, a methanotroph isolated from Hardangervidda national park (Norway). This strain represents the second of the two validly published species genus with a sequenced genome. The other is *M. miyakonense* HT12, which is the type strain of the species and the type species of the genus *Methylovulum.* We present the genome of *M. psychrotolerants* str. HV10-M2 and discuss the differences between *M. psychrotolerans* and *M. miyakonense*. The genome size of *M. psychrotolerans* str. HV10-M2 is 4,923,400 bp and contains 4415 protein-coding genes, 50 RNA genes and an average GC content of 50.88%.

**Electronic supplementary material:**

The online version of this article (10.1186/s40793-018-0314-2) contains supplementary material, which is available to authorized users.

## Introduction

Methanotrophs are a group of microorganisms that utilize methane as the sole energy and carbon source. They are important contributors to the global carbon budget and climate change, reducing methane emissions to the atmosphere as they represent the only known biological methane sink [1]. Aerobic methane oxidation can be performed by members of the phyla *Proteobacteria* (classes *Alphaproteobacteria* and *Gammaproteobacteria*) and *Verrucomicrobia* [2, 3]. The recently described genus *Methylovulum* [4] belongs to the family *Methylococcaceae* within the class *Gammaproteobacteria*. Their cells are described as aerobic, non-motile gram-negative bacteria with coccoid or rod-shape cells, they grow with methane and methanol as carbon sources and they have been isolated from cold environments [5]. So far, only one species within this genus, *Methylovulum miyakonense* HT12, has published available genome data. However, the 16S rRNA gene sequence of three isolates belonging to the *Methylovulum* genus have been recently reported [5].

**Fig. 1:**
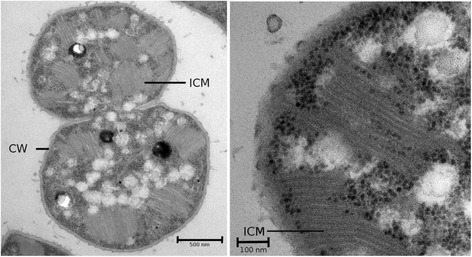
Transmission electron microscope picture of the strain *Methylovulum psychrotolerans* HV10-M2. Cell wall (CW) and intracytoplasmatic membrane (ICM) are labelled in the pictures. Scale bars represent 500 (left panel) and 100 (right panel) nm

Here we report the characteristics of *M. psychrotolerans* HV10-M2 (Fig. 1), isolated from plant material collected from a peat bog saturated with water at Hardangervidda, a high-altitude (> 1230 m above sea level) national park located in central Norway. We present the genome of *M. psychrotolerans* str. HV10-M2, and provide first insights into the genomic and physiological differences between *M. psychrotolerans* HV10-M2 and *M. miyakonense* HT12.

## Organism information

### Classification and features

*M. psychrotolerans* HV10-M2 was isolated from wet plant material located in a peat bog at the high-altitude (1230 m above sea level) national park Hardangervidda (Norway) (GPS: 60.22 N, 7.25 E) on July 17, 2015. Air temperature during sampling was 9 °C. The strain was enriched from approximately 4 g of the plant material collected from the peat bog which was added directly to a 120 mL sterile serum flask containing 20 mL of LMM medium (Low-Salt-Methanotrophic medium; KNO_3_ 0.1 g L^− 1^, MgSO_4_ 0.1 g L^− 1^, CaCl_2_·2H_2_O 0.02 g L^− 1^, KBr 0.01 g L^− 1^ [6]), closed with a butyl rubber stopper and sealed with an aluminum crimp. Methane (purity 99.5%, Yara Praxair, Oslo, Norway) was amended with a syringe through a 0.2 μm DynaGard® filter (CA, USA) to a final ratio of 4:1 (methane/air; *v*/v). The flask was incubated under dark conditions at 16 °C for three weeks without shaking. The gas mixture was restored every seven days, and growth was monitored using phase contrast microscopy (Eclipse E400 microscope, Nikon Corporation, Tokyo, Japan).

The enrichment culture was transferred five times into serum flasks with LMM medium as described above. Serial dilutions (10^− 5^ to 10^− 8^) were then prepared and aliquots of 0.1 mL of each dilution were spread onto LMM agar plates. Plates were incubated at 16 °C in jars filled with a methane:air gas mixture (4:1, v/v). Single pink colonies (previously isolated *M. psychrotolerans* strains show pink pigmentation [5]) were picked and re-streaked onto new agar plates. Finally, one single colony was transferred into a serum flask with LMM medium (see above) and incubated for one week at 16 °C. Purity of the isolate was confirmed by PCR and transmission electron microscopy (TEM, at 60 kV, Jeol JEM-1230, Tokyo, Japan). Contamination was assessed as reported previously [6, 7].

*M. psychrotolerans* HV10-M2 grows between 4 °C and 25 °C, with optimal growth between 13 °C to 20 °C. Strain HV10-M2 grows using methane and methanol as the carbon and energy source. The optimal pH for growth 6.8. Cells of HV10-M2 are aerobic, non-motile, coccoid- to rod-shaped and form light pink colonies when checked on LMM agar plates. Average cell size is 2 μm. The characteristics of *M. psychrotolerans* HV10-M2 are summarized in Table 1.

**Table 1:** Classification and general features of *Methylovulum psychrotolerans* strain HV10_M2^T^

MIGS ID	Property	Term	Evidence code^a^
	Classification	Domain *Bacteria*	TAS [34]
		Phylum *Proteobacteria*	TAS [35]
		Class *Gammaproteobacteria*	TAS [36]
		Order *Methylococcales*	TAS [37]
		Family *Methylococcaceae*	TAS [38]
		Genus *Methylovulum*	TAS [4]
		Species *psychrotolerans*	TAS [5]
		Strain: *Sph1*	TAS [5]
	Gram stain	*Negative*	IDA
	Cell shape	*Coccoid / Rod-shape*	IDA
	Motility	*Non-motile*	IDA
	Sporulation	*Non-sporulating*	IDA
	Temperature range	*4–25 °C*	IDA
	Optimum temperature	*13–20 °C*	IDA
	pH range; Optimum	*5–7; 6.8*	IDA
	Carbon source	*Methane*	IDA
MIGS-6	Habitat	*Peat bog*	IDA
MIGS-6.3	Salinity	ND	–
MIGS-22	Oxygen requirement	*Aerobic*	IDA
MIGS-15	Biotic relationship	*Free-living*	IDA
MIGS-14	Pathogenicity	*Non-pathogen*	NAS
MIGS-4	Geographic location	*Hardangervidda, Norway*	IDA
MIGS-5	Sample collection	*2015*	IDA
MIGS-4.1	Latitude	*60.22 N*	IDA
MIGS-4.2	Longitude	*7.25 E*	IDA
MIGS-4.4	Altitude	*1230 m a.s.l.*	IDA

^a^Evidence codes - *IDA* Inferred from Direct Assay, *TAS* Traceable Author Statement (i.e., a direct report exists in the literature), *NAS* Non-traceable Author Statement (i.e., not directly observed for the living, isolated sample, but based on a generally accepted property for the species, or anecdotal evidence). These evidence codes are from the Gene Ontology project [39]

**Fig. 2:**
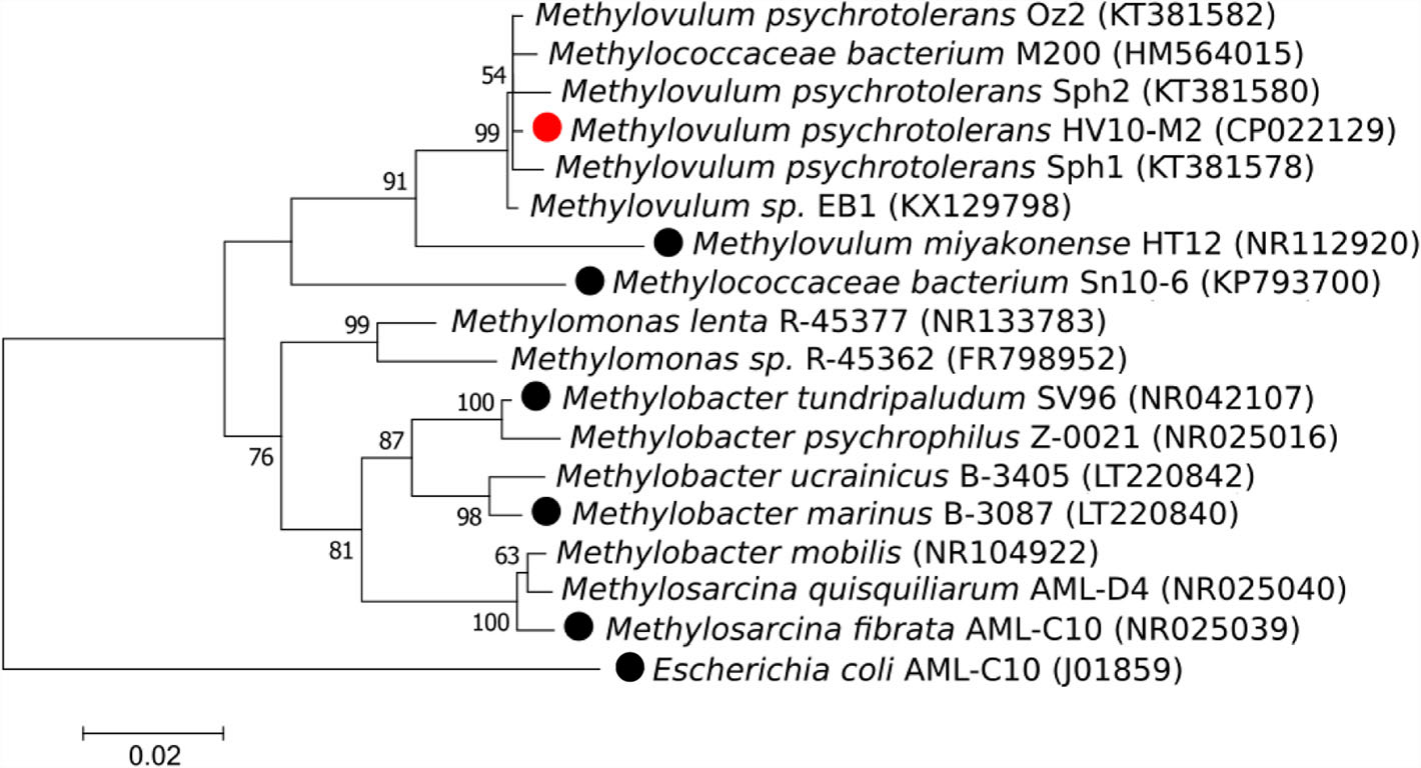
Phylogenetic tree. The tree is based on the 16S rRNA gene sequences of the best hits of cultured strains in the nucleotide database from NCBI (retrieved on June 5, 2017). The tree was reconstructed by using maximum likelihood analysis and the Jukes-Cantor nucleotide substitution model as implemented in MEGA v7 [40]. Robustness of the tree was determined using 1000 bootstrap replicates. Sequences were aligned with MUSCLE [41] in MEGA v7. The tree was rooted against the 16S rRNA gene sequence of *Escherichia coli*. Accession numbers are provided within parenthesis. The strain presented in this study is marked with a red dot. Strains with available genome data are marked with black dots. Bootstrap values less than 50 are not shown

The 16S rRNA gene of *M. psychrotolerans* HV10-M2 shows more than 99% sequence identity with *M. psychrotolerans* Sph1, Sph2 and Oz2 (GenBank accession numbers KT381578, KT381580 and KT381582, respectively; Fig. 2). However, none of those strains has genome data publicly available. The highest sequence identity with a strain with genome data was *M. miyakonense* HT12. The two strains *M. psychrotolerans* HV10-M2 and *M. miyakonense* HT12 show 95% sequence identity in the 16S rRNA gene (Fig. 2).

## Genome sequencing information

### Genome project history

*M. psychrotolerans* HV10-M2 was whole genome sequenced at the Department of Bioscience at Aarhus University, Denmark in September 2016. The genome project was deposited in GOLD under the project Ga0185950. The Whole Genome Shotgun project was deposited at GenBank with the accession number CP022129. Summarized information about the project and the sequencing platform details are included in Table 2.

**Table 2:** Project information

MIGS ID	Property	Term
MIGS 31	Finishing quality	High-quality-draft
MIGS-28	Libraries used	Paired-end NexteraXT DNA
MIGS 29	Sequencing platforms	Illumina MiSeq
MIGS 31.2	Fold coverage	303.7X
MIGS 30	Assemblers	SPAdes 3.9.0
MIGS 32	Gene calling method	Prodigal v2.6.2
	Locus Tag	CEK71
	Genbank ID	CP022129
	GenBank Date of Release	2017–06-27
	GOLD ID	Gp029646
	BIOPROJECT	PRJNA391059
MIGS 13	Source Material Identifier	HV10-M2
	Project relevance	Environmental

### Growth conditions and genomic DNA preparation

*M. psychrotolerans* HV10-M2 was cultivated in a 120 ml serum flask at 16 °C containing LMM medium with methane addition. After turbidity was observed (approximately 2 months), it was transferred onto LMM agar plates and incubated with methane as headspace gas (4:1, *v*/v). A single colony was transferred into a flask containing LMM medium and finally, 1.5 mL of the culture was harvested by centrifugation and genomic DNA was isolated from the pellet using the GenElute Bacterial Genomic DNA kit (Sigma Aldrich, USA) following the manufacturer recommendations and purified using the DNA Clean and Concentrator kit (Zymo Research, USA).

### Genome sequencing and assembly

The genomic DNA of strain HV10-M2 was sequenced with the Illumina MiSeq Reagent Kit V3 (Illumina, CA, USA) and the sequencing libraries were prepared using the Nextera XT Library Preparation Kit (Illumina). A total of 4,933,624 sequence reads were generated. FastQC [8] was used for quality control. Reads were adaptor- and quality trimmed using Trimmomatic v0.36 [9] when bases were at the end of the reads and when the average quality was below the quality threshold (Phred score < 33) with the parameters: CROP:289, HEADCROP:19, SLIDINGWINDOW:4:20, MINLEN:100, resulting in 4,019,650 paired-end reads and 4.92 Mb with an overall coverage estimate of 303.7×. Assembly of the data was performed using SPAdes v3.9.0 [10] with the “--careful” option and the *k*-mer values 21, 33, 55, 77, 99, 127. The assembly was evaluated with QUAST v4.3 [11]. The assembly yielded 186 contigs with a total length of 4,923,400 bp, and an N_50_ value of 71,358 bp.

### Genome annotation

Gene prediction and annotation was performed using the standard operating procedure of the Integrated Microbial Genomes platform developed by the Joint Genome Institute [12]. In addition, the genome of *M. psychrotolerans* HV10-M2 was submitted to BlastKOALA [13] and Pathway tools [14] to be compared against the KEGG [15] and MetaCyc [16] databases, respectively, for pathway predictions.

## Genome properties

The properties of the draft genome of *M. psychrotolerans* HV10-M2 are shown in Table 3 and the genes associated with COG functional categories in Table 4. The genome consists of 4,923,400 bp with a GC content of 50.88%. The genome is estimated to be 99% complete as determined by CheckM v1.0.7 [17] compared to the family *Methylococcaceae*. In total 4465 genes were predicted: 50 RNA genes and 4415 protein-coding genes. In addition, 2344 genes were assigned in COG functional categories. The PHAST program, used to detect prophages sequences in bacterial genomes [18], determined no evidence of completed prophages in the genome of *Methylovulum psychrotolerans* HV10-M2.

**Table 3:** Genome statistics

Attribute	Value	% of Total
Genome size (bp)	4,923,400	100
DNA coding (bp)	4,194,869	85.20
DNA G + C (bp)	2,504,955	50.88
DNA scaffolds	186	100
Total genes	4465	100
Protein coding genes	4415	98.88
RNA genes	50	1.12
Pseudo genes	0	0
Genes in internal clusters	804	18.01
Genes with function prediction	2984	66.83
Genes assigned to COGs	2344	52.50
Genes with Pfam domains	3159	70.75
Genes with signal peptides	438	9.81
Genes with transmembrane helices	897	20.09
CRISPR repeats	0	0

**Table 4:** Number of genes associated with general COG functional categories

Code	Value	%age	Description
J	194	7.48	Translation, ribosomal structure and biogenesis
A	2	0.08	RNA processing and modification
K	124	4.78	Transcription
L	121	4.66	Replication, recombination and repair
B	1	0.04	Chromatin structure and dynamics
D	40	1.54	Cell cycle control, Cell division, chromosome partitioning
V	88	3.39	Defense mechanisms
T	182	7.01	Signal transduction mechanisms
M	231	8.9	Cell wall/membrane biogenesis
N	56	2.16	Cell motility
U	56	2.16	Intracellular trafficking and secretion
O	137	5.28	Posttranslational modification, protein turnover, chaperones
C	161	6.2	Energy production and conversion
G	97	3.74	Carbohydrate transport and metabolism
E	160	6.17	Amino acid transport and metabolism
F	63	2.43	Nucleotide transport and metabolism
H	156	6.01	Coenzyme transport and metabolism
I	68	2.62	Lipid transport and metabolism
P	154	5.93	Inorganic ion transport and metabolism
Q	45	1.73	Secondary metabolites biosynthesis, transport and catabolism
R	243	9.36	General function prediction only
S	148	5.7	Function unknown
–	2121	47.5	Not in COGs

The total is based on the total number of protein coding genes in the genome

## Insights from the genome sequence

Here we present the draft genome sequencing and annotation of *M. psychrotolerans* HV10-M2. The 16S rRNA gene of *Methylovulum psychrotolerans* HV10-M2 shows a 99% sequence identity with *M. psychrotolerans* strains Sph1, Sph2 and Oz2 as well as *Methylovulum* *sp.* Eb1 and *Methylococcaceae* *bacterium* M200 (see also Fig. 2). The highest identity to a strain with an available genome sequence was 95% with *Methylovulum miyakonense* HT12, which is the only genome sequenced species within the *Methylovulum* genus.

### Extended insights

Methanotrophic microorganisms oxidize methane to carbon dioxide and water. In a first step, methane is converted into methanol in a process catalyzed by the methane monooxigenase enzyme (MMO). There are two types of MMO, the membrane-bound particulate MMO (pMMO), which is found in all methanotrophs except for some members of the genera *Methylocella* and *Methyloferula* [19, 20], and the cytoplasmatic soluble MMO (sMMO), which is limited to very few species [21]. In the genome of *M. psychrotolerans* HV10-M2, the presence of a single copy of the gene cluster *pmo*ABC was observed (locus tags RS17435, RS17440, RS17440). The *pmo*ABC cluster contains the genes encoding for the pMMO. However, the *mmoX* gene encoding for the sMMO was absent in the genome of *M. psychrotolerans* HV10-M2. The *mmoX* gene is reported to be present in *M. miyakonense* HT12. To confirm the absence of this gene in *M. psychrotolerans* HV10-M2, the *mmoX* gene sequence was blasted against the genome of *M. psychrotolerans* HV10-M2 using the sequence of *M. miyakonense* HT12 as query (Genbank accession number AB501287). In addition, a *mmoX*-specific PCR with the primer set 882F/1403R [22] was performed. Both approaches confirmed the absence of the *mmoX* gene in *M. psychrotolerans* HV10-M2. Additionally, the *pxmABC* operon described in some gammaproteobacterial methanotrophs [23], was not observed in the genome of *M. psychrotolerans* HV10-M2.

The following step in the oxidation of methane is the conversion of methanol into formaldehyde. This reaction is catalyzed by a methanol dehydrogenase that contains a pyrroloquinoline quinone as a cofactor and requires a cytochrome *c* as electron acceptor [24]. The gene clusters associated with this step present in the genome of *M. psychrotolerans* HV10-M2 are: (i) the gene cluster *mxa*FJ that encodes the components active in methanol oxidation (locus tags RS12435, RS12440); (ii) the cluster *mxa*ACKL required for MDH synthesis and PQQ insertion into the MDH (locus tags RS12465, RS12475, RS12480); and (iii) the gene cluster *pqq*ABCDE involved in the PQQ biosynthesis (locus tags RS20845, RS01900, RS01880, RS05860, RS12150). The MDH enzyme is also present in *M. miyakonense* HT12. The gene *xoxF* that encodes for a polypeptide with similar sequence to the MxaF protein, has been suggested as an alternative to the MDH [25]. High identity (89%) between a gene encoding for a PQQ-dependent dehydrogenase in *M. psychrotolerans* HV10-M2 (locus tag RS12435) and a methanotrophic XoxF protein (Accesion number: SAJ59293), suggesting that this protein could be also present in *M. psychrotolerans* HV10-M2.

The next step is the formaldehyde oxidation. This step is crucial, as formaldehyde is a cytotoxic compound. The methanotrophs can use different pathways to perform oxidation of formaldehyde to formate. In the tetrahydromethanopterin (H_4_MPT)-linked C_1_ transfer pathway, presence of the genes encoding for the tetrahydromethanopterin protein and processes such as *fae*, *mch* and *mtd*B, were observed in the genome of *M. psychrotolerans* HV10-M2. Most likely the H_4_MPT-linked pathway will act as a secondary pathway involved in formaldehyde assimilation as the Ribulose MonoPhosphate will act as the principal pathway [24]. In addition, like in most of the methylotrophs, presence of the genes encoding for the tetrahydrofolate pathway including the methylene-H_4_F dehydrogenase and methenyl-H_4_F cyclohydrolase enzymes of the FoID [24], are also present in the genome of *M. psychrotolerans* HV10-M2 (locus tags RS10730, RS15610).

Methanotrophic microorganisms can be divided into type I (*Gammaproteobacteria*) and type II (*Alphaproteobacteria*) based on the cyclic pathway followed to perform C_1_ assimilation. Recently, type X methanotrophs have also been described [26]. Type I methanotrophs, such as *M. psychrotolerans* HV10-M2, use the RuMP cycle, whereas type II methanotrophs use the serine cycle. Briefly, in the first step of the RuMP pathway D-arabino-3-hexulose-6-phosphate is formed from ribulose-5-phosphate, which will be later converted into fructose-6-phosphate. Then, fructose-6-phosphate is converted into Fructose-1,6-bisphosphate through the 6-phosphofructokinase using PP_i_ as the donor in a reversible reaction. Finally, an aldolase will form glyceraldehyde-3-phosphate.

Several genes encoding for enzymes used in the Serine cycle such as hydroxytransmethylase, serine-glyoxylate aminotransferase and hydroxypyruvate reductase were also present in the genome of *M. psychrotolerans* HV10-M2. However, the serine cycle is not completed, as the genes encoding for the malyl-CoA lyase enzyme could not be found in the genome. This is not surprising as other type I methanotrophs such as *M. miyakonense* HT12, *Methylobacter tundripaludum* SV96 [27] or *Methylomicrobium album* BG8 [28] also contain the genes encoding for most of the serine pathway. However, in the latter two, the phosphoenolpyruvate carboxylase enzyme is absent in the genome while in the *Methylovulum* strains is present. So far, there is no knowledge about any methanotrophic strain encoding for all the enzymes in both, the RuMP and the serine cycle.

The oxidation of formate to CO_2_ is performed by the formate dehydrogenase enzyme. The genes encoding for the FDH enzyme were also present in the genome of *M. psychrotolerans* HV10-M2 (locus tag RS07700). It has been previously reported that this step is less demanding in organisms using the RuMP pathway for formaldehyde assimilation, such as *M. psychrotolerans* HV10-M2, and therefore FDH activities are very low [24].

It is known that some methanotrophs such as members of the genera *Methylococcus* and *Methylocaldum* (Type X methanotrophs), have genes encoding for enzymes involved in the Calvin-Benson-Basham cycle responsible for carbon dioxide fixation [29]. However, no evidence of the genes encoding for the ribulose-1,5-biphosphate carboxylase/oxygenase (RuBisCO), *cbb*L, *cbb*S and *cbb*Q, was found in the genome of *M. psychrotolerans* HV10-M2.

Methanotrophic bacteria also play a major role in the nitrogen cycle. The MMO can oxidize ammonia into nitrite and nitrous oxide as they are evolutionary related [30]. The prevailing view was that only type II and type X methanotrophs could have the ability to fix nitrogen, and although recently it has been suggested that some type I methanotrophs (*Methylomonas* and *Methylobacter*) could also perform nitrogen assimilation. Although in the genome of *M. psychrotolerans* HV10-M2 the nitrogenase gene cluster *nif*DKH was present (locus tag RS1055, RS01060, RS01050), the *anf*G gene encoding for the nitrogenase delta subunit was absent, therefore *M. psychrotolerans* HV10-M2 cannot fix nitrogen. This result matches with other members of the genus *Methylovulum* where no growth in nitrogen-free medium was reported [4]. The nitrite reductase genes *nirK* and *nirS* involved in the formation of nitric oxide were not found in the genome of *M. psychrotolerans* HV10-M2, furthermore the genes *norBC*, involved in the following reaction (formation of nitrous oxide from nitric oxide), were not detected in the genome of *M. psychrotolerans* HV10-M2. Although the nitrite reductase genes are common in *Gammaproteobacteria* methanotrophs, they have not been found in others such as *M. miyakonense* HT12 or *Methylomicrobium alcaliphilum* or *Methylomonas* *denitrificans* [4, 31]. The absence of the genes *norBC*, present in the latter methanotrophs, in *M. psychrotolerans* HV10-M2 could be due to the completeness of the genome. Additionally the presence of the *haoAB* genes, encoding for the hydroxylamine dehydrogenase, that have been observed to be variable in *Gammaproteobacteria* methanotrophs, are absent in the genome of *M. miyakonense* HT12 and *M. psychrotolerans* HV10-M2.

Compared to the reference strain of the *Methylovulum* genus, *Methylovulum miyakonense* HT12, there are genetic and morphological differences with *M. psychrotolerans* HV10-M2. The former has a pink color while the latter exhibited brown color. Additionally, the sMMO enzyme has been found only in one strain within the *Methylovulum* genus (*M. miyakonense* HT12). Further, the optimal growth temperature range between *M. psychrotolerans* and *M. miyakonense* is different. *M. psychrotolerans* is psychrotolerant with an optimal growth temperature between 13 and 20 °C, whereas *Methylovulum miyakonense* HT12 is mesophilic with an optimal temperature between 24 and 32 °C. Interestingly, most of the characteristics not shared between those strains such as, the pink color and the absence of the sMMO enzyme, are common within the *M. psychrotolerans* strains including the strain M200 in the family *Methylococcaceae* [32] (Information about *Methylovulum* *sp*. Eb1 is not available).

Those differences together with the 95% identity in the 16S rRNA gene between *M. miyakonense* HT12 and *M. psychrotolerans* HV10-M2 could suggest that the former belong to a different genus. To investigate this, the average nucleotide identity using BLAST were performed with the draft genomes. The strains shared ANIb values of 79.2%, being the highest amongst the closest strains with genome data available (Additional file 1: Table S1). Furthermore, recently it has been proposed that a prokaryotic genus can be defined as a group of species with pairwise values in the percentage of conserved proteins higher than 50% [33]. The POCP value between *M. miyakonense* HT12 and *M. psychrotolerans* HV10-M2 was 62.9%, therefore suggesting that *M. miyakonense* HT12 and *M. psychrotolerans* HV10-M2 belong to the same genus.

## Conclusions

In the present study, we present the high-quality draft genome of *Methylovulum psychrotolerans* HV10-M2. The genome consists of 4,923,400 bp in 4415 protein-coding genes, 50 RNA genes with and an average 50.88% GC content. As the *Methylovulum* genus has been recently described [4] only one genome has been available so far and this is from *Methylovulum miyakonense* HT12. *M. psychrotolerans* HV10-M2 has a 95% sequence identity with *M. miyakonense* HT12. In addition, there are some differences between both species, such as the *mmoX* gene, which encodes for the sMMO enzyme. The *mmoX* gene is present only in *M. miyakonense* whereas in *M. psychrotolerans* is absent. The other differences are the colour, as *M. miyakonense* showed a brown colour and *M. psychrotolerans* is pink, and the optimal growth temperature. *M. miyakonense* is mesophilic and *M. psychrotolerans* is psychrotolerant.

## Additional file

Additional file 1: **Table S1.** ANIb analysis results with the similarities between the draft genomes of the four closest strains to M. psychrotolerans HV10-M2. (DOCX 15 kb)

## Abbreviations

CBB: Calvin-Benson-Basham
FDH: Formate dehydrogenase
H_4_F: Tetrahydrofolate
H_4_MPT: Tetrahydromethanopterin
LMM: Low-salt methanotrophic medium
MDH: Methanol dehydrogenase
MMO: Methane monooxigenase
pMMO: Membrane-bound particulate MMO
POCP: Percentage of conserved protein
PQQ: Pyrroloquinoline quinone
RuBisCO: Ribulose-1,5-biphosphate carboxylase/oxygenase
RuMP: Ribulose MonoPhosphate
sMMO: cytoplasmatic soluble MMO
