# Supplementary material for: High-quality draft genome of the methanotroph Methylovulum psychrotolerans Str. HV10-M2 isolated from plant material at a high-altitude environment
Source: Stand Genomic Sci. 2018 Apr 12;13:10. doi: 10.1186/s40793-018-0314-2 (PMC5898042; doi:10.1186/s40793-018-0314-2)
Supplement: Supplementary file 1 — Table S1. ANIb analysis results with the similarities between the draft genomes of the four closest strains to M. psychrotolerans HV10-M2. (DOCX 15 kb) [file 40793_2018_314_MOESM1_ESM.docx]

**Additional file:**

**Table S1.** ANIb analysis results of the four closest strains to *M. psychrotolerans* HV10-M2.

| **Strain no.** | **Strain name** | **% Similarity with strain** | | | | |
| --- | --- | --- | --- | --- | --- | --- |
|  |  | **1** | **2** | **3** | **4** | **5** |
| **1** | *Methylovulum psychrotolerans* HV10-M2 |  | 79.21 | 76.33 | 76.32 | 75.62 |
| **2** | *Methylovulum miyakonense* HT12 | 79.21 |  | 76.75 | 76.65 | 75.67 |
| **3** | *Methylobacter tundripaludum* SV96 | 76.33 | 76.75 |  | 77.69 | 75.94 |
| **4** | *Methylobacter marinus* | 76.32 | 76.65 | 77.69 |  | 75.43 |
| **5** | *Methylococcaceae bacterium* Sn10-6 | 75.62 | 75.67 | 75.94 | 75.43 |  |
